# Sexually transmitted founder HIV-1 viruses are relatively resistant to Langerhans cell-mediated restriction

**DOI:** 10.1371/journal.pone.0226651

**Published:** 2019-12-19

**Authors:** Nina Hertoghs, Bernadien M. Nijmeijer, Nienke H. van Teijlingen, Angharad E. Fenton-May, Tanja M. Kaptein, John L. van Hamme, John C. Kappes, Neeltje A. Kootstra, Beatrice H. Hahn, Persephone Borrow, Carla M. S. Ribeiro, Teunis B. H. Geijtenbeek

**Affiliations:** 1 Department of Experimental Immunology, Amsterdam Infection and Immunity Institute, Amsterdam University Medical Centers, University of Amsterdam, Amsterdam, the Netherlands; 2 Nuffield Department of Clinical Medicine, University of Oxford, Oxford, United Kingdom; 3 Department of Medicine, University of Alabama at Birmingham, Birmingham, AL, United States of America; 4 Departments of Medicine and Microbiology, University of Pennsylvania, Philadelphia, PA, United States of America; Scripps Research Institute, UNITED STATES

## Abstract

A single HIV-1 variant establishes infection of the host after sexual contact. Identifying the phenotypic characteristics of these Transmitted Founder (T/F) viruses is important to understand the restriction mechanisms during transmission. Langerhans cells (LCs) are the mucosal dendritic cell subset that has been shown to have a protective role in HIV-1 transmission. Immature LCs efficiently capture and degrade HIV-1 via langerin-mediated restriction. Here we have investigated the capacity of T/F HIV-1 strains to infect mucosal Langerhans cells (LCs). Notably, most T/F variants efficiently infected immature LCs derived from skin and vaginal tissue in contrast to chronic HIV-1 laboratory strains. Next we screened a panel of T/F viruses and their matched 6-month consensus sequence viruses. Interestingly most T/F variants infected immature LCs whereas donor-matched 6-month consensus sequence viruses had lost the ability to infect LCs. However, we also identified 6-month consensus sequence viruses that had retained an ability to infect LCs similar to that of the donor-matched T/F virus. Moreover, some T/F viruses and 6-month consensus sequence viruses were unable to infect immature LCs. Further analyses indicated that T/F viruses are less sensitive to langerin-mediated restriction. These data suggest that T/F HIV-1 variants have the ability to infect immature LCs, which will facilitate transmission.

## Introduction

At the time of sexual transmission, different HIV-1 variants are present in seminal fluid and vaginal secretions, but typically only a single viral genotype establishes a new infection. These so-called Transmitted Founder (T/F) viruses are thought to have specific properties that allow them to become transmitted to the host. Study of these phenotypes helps to provide a thorough understanding of the interplay between the local restriction mechanisms and HIV-1 transmission.

During sexual transmission, Langerhans cells (LCs) are the first immune cells to encounter HIV-1 and are thought to act as a barrier to HIV-1 as LCs are highly refractory to HIV-1 infection [1–3]. Immature LCs do not become infected by HIV-1 and the C-type receptor (CLR) langerin on LCs restricts HIV-1 infection via TRIM5α-induced autophagosomal degradation of HIV-1 [2]. Therefore, LCs form a protective barrier against HIV-1 and normally prevent virus dissemination to CD4+ T-cells. However, immune activation or langerin inhibition allows infection of LCs, which subsequently transmit HIV-1 to CD4+ T cells [4]. Nothing is known about the susceptibility of LCs to infection by T/F-viruses. Here, we have investigated their ability to infect LCs derived from vaginal mucosa as well as skin *in vitro* and *ex vivo*.

Notably, LCs were more efficiently infected by T/F-viruses than laboratory-adapted HIV-1 strains, whereas, in contrast, monocyte-derived dendritic cells (moDCs) were less susceptible to T/F-viruses than laboratory strains. Interestingly, T/F-viruses were less sensitive to langerin-mediated restriction. Thus, our data strongly suggest that the ability to infect LCs might be important in the establishment of infection by T/F-viruses. Understanding the mechanism underlying the escape from langerin-restriction by HIV-1 will help to develop ways to harness LCs in protection against HIV-1 infection.

## Materials and methods

### Ethics statement

Studies using human skin tissue was done in accordance with our institutional guidelines with approval of the Medical Ethics Review Committee of the Amsterdam University Medical Centers, location Academic Medical Center (AMC), Amsterdam, the Netherlands, reference number: W15_089 # 15.0103. The studies concerning vaginal tissue was approved by the Medical Ethics Review Committee of the Amsterdam University Medical Centers, location AMC, reference number: W13_046 # 13.17.0060. Written informed consent was obtained. Buffy coats obtained after blood donation (Sanquin blood bank, the Netherlands) is not subjected to informed consent according to the Medical Research Involving Human Subjects Act and the Medical Ethics Review Committee of Amsterdam University Medical Centers, location AMC. All samples of skin tissue, vaginal tissue and buffy coats were handled anonymously.

### Antibodies and reagents

The following antibodies were used: human CD1a-APC, mouse IgG1 monoclonal (#559775) (BD Biosciences, USA), human CD207-PE, mouse IgG1 monoclonal (#IM3577) (BeckmanCoulter, USA) and human anti-HIV-1 p24-PE, (#KC57-RD1), mouse IgG1 monoclonal (Beckman Coulter, USA).). The following reagents were obtained through the NIH AIDS Reagent Program, NIAID: Zidovudine (AZT), Raltegravir (RAL) and Indinavir (IDV).

### Primary cell isolation and cell lines

The isolation of monocytes from buffy coats of healthy donors (Sanquin blood bank) and differentiation into immature DCs and the subsequent phenotyping was done as described before [5,6].

Human skin tissue LCs were obtained from healthy donors undergoing corrective surgery. LC-enriched epidermal single-cell suspensions were generated as described before [1–3]. Briefly, epidermal sheets were incubating in PBS containing DNase I (20 units/ml; Roche Applied Science) and trypsin 0.05% (Beckton Dickinson, USA). Single-cell suspension was layered on Ficoll gradient (Axis-shield) and immature LCs were purified using CD1a microbeads (Miltenyi Biotec, Germany). LCs were routinely 90% to 98% pure and expressed high levels of langerin and CD1a [1,3]. Activated LCs were generated as described before [1]. Briefly, epidermal sheets were cultured in IMDM (Thermo Fischer Scientific, USA) supplemented with 10% FCS, gentamycine (20 μg/ml, Centrafarm, Netherlands), pencilline/streptomycin (10 U/ml and 10 μg/ml, respectively; Invitrogen) for 3 days and activated LCs were harvested.

Vaginal tissue was obtained from healthy donors undergoing vaginal prolapse surgery and were separated from the underlying lamina propria and submucosa after overnight incubation with dispase II (Roche Diagnostics). A single cell suspension was obtained by mechanical fragmentation of the epithelial sheets and subsequent incubation with trypsin (0,05%, Invitrogen) and DNase I (100 U/mL, Roche diagnostics). Further purification was also performed using a Ficoll gradient and a positive selection with CD1a microbeads. The U87 cell line was obtained through the NIH AIDS Reagent Program, Division of AIDS, NIAID, NIH: U87 CD4^+^CCR5^+^ cells from H. K. Deng and D. R. Littman [7]. The langerin-expressing U87 cells were generated as described before [2].

### Viruses

Viruses were produced by transfection of 293T cells by using Genejuice transfection reagent (Novagen) as described [4]. A panel of T/F plasmids were obtained through the NIH AIDS Reagent Program, Division of AIDS, NIAID, NIH: Panel of full-length transmitted/founder (T/F) HIV-1 Infectious Molecular Clones (Cat #11919) from Dr. John Kappes [8–10]. Plasmids were amplified by transformation into STBL3 *E*.*coli* bacteria (Invitrogen). For plasmid purification, the nucleobond XtraMidi (Macherey-Nagel) was used and the size of the plasmids was checked by restriction analysis. After transfection, the concentration of the produced viruses was quantified by a p24 antigen ELISA (Perkin Elmer Life Sciences), and the viruses were titrated using the indicator cells TZM-bl (John C. Kappes, Xiaoyun Wu, Birmingham, Alabama, USA and TranzymeInc.), the NIH AIDS Reagent Program [11]. For infections, the viruses were normalized based on this titration. Infectious molecular clones of five T/F and patient-matched 6-month (6-mo) consensus sequence virus pairs were generated and viral stocks produced as previously described [12,13]. The sequences for CH164 6-mo and CH042 6-mo have been deposited in the genbank: KC156128 and KC156124 respectively. Viral titers were measured by reverse transcriptase assay (Roche Applied Science).

### Infection

Immature, activated and vaginal LCs and moDCs were infected at an MOI of 0.05–0.1 for 5 days. For comparison between T/F viruses and their respective 6-month consensus sequence pairs, immature LCs were infected with 25ng/ml RT activity and harvested after 5 days. *Ex vivo* sheets were directly infected, and after 3 days emigrated LCs were collected, washed and cultured for 2 days. Infection and expression of relevant markers were assessed by using antibodies against CD1a (1;25), CD207 (1:25) and p24 (1:200) and flow cytometric analysis was done using a FACSCanto II flow cytometer (BD Biosciences). The analysis of the data was performed with FlowJo software (Treestar) and Graphpad (Prism).

### Statistical analysis

Statistical significance for moDCs, immature, activated and vaginal LCs was determined using an unpaired, non-parametric, Mann-Whitney test. A two-tailed Student’s t-test for unpaired observations was used to determine statistical significance for comparing infection of different U87 cell lines. Statistical analyses were performed using GraphPad 7.0 software and significance was set at P < 0.05 (* P < 0.05; ** P < 0.01, *** P <0.001, **** P <0.0001), ns = not significant.

## Results

### Langerhans cells but not dendritic cells are more susceptible to T/F viruses

**Fig 1:**
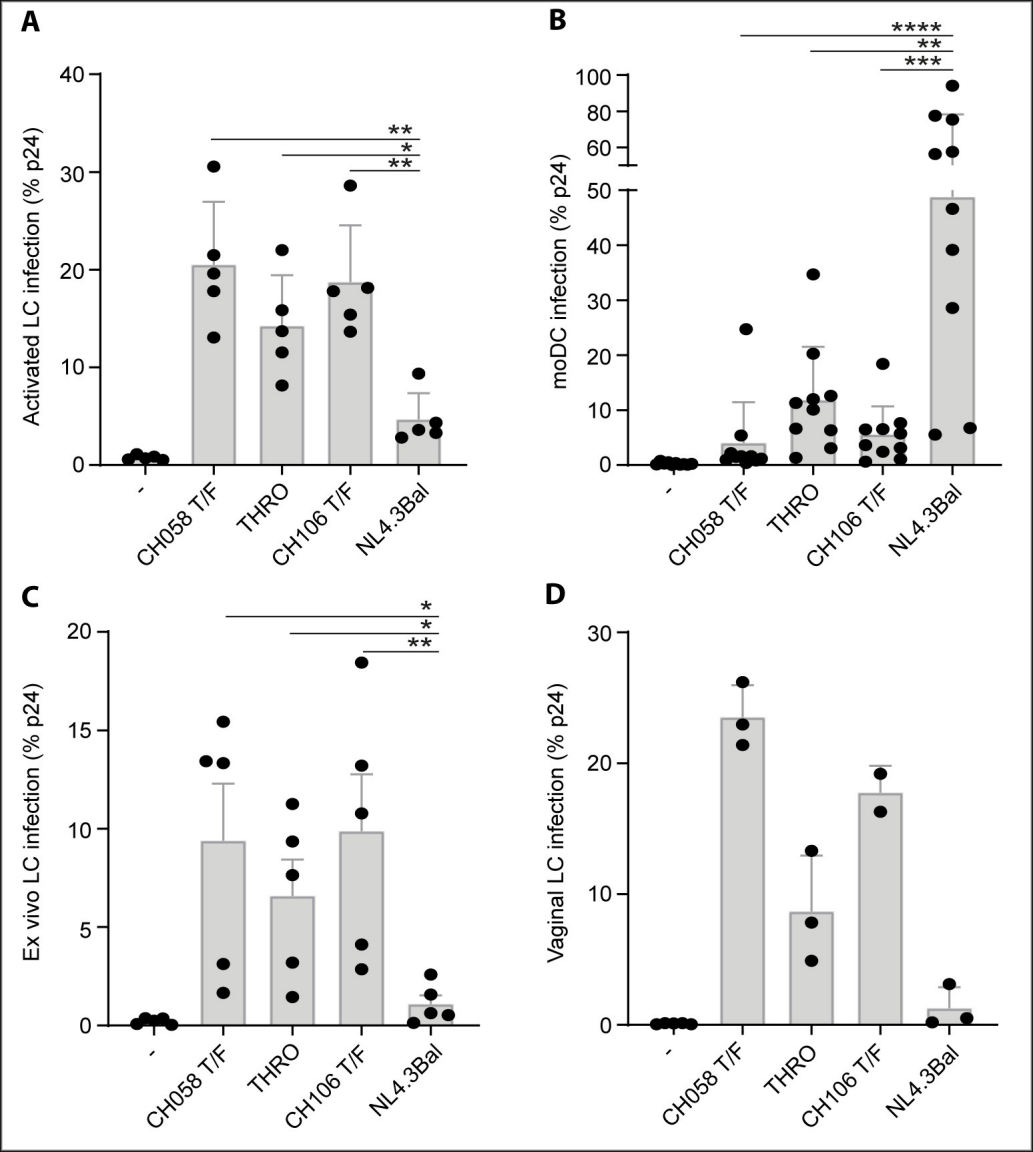
Langerhans cells but not dendritic cells are more susceptible to T/F viruses. (A-B) Infection of (A) activated LCs and (B) moDCs by T/F-viruses and NL4.3Bal. (C) Ex vivo tissue explant was infected by different T/F viruses and NL4.3Bal and infection of emigrated LCs was measured. (D) Infection of vaginal immature LCs by different T/F-viruses and NL4.3Bal. The viral titers were normalized based on TCID50 measurements. Each dot represents the average percentage of infection per donor. Error bars are the mean ± SD of A, n = 5; B, n = 10; C, n = 5; D, n = 3 donors. LC: Langerhans cell, moDC: monocyte-derived dendritic cell, T/F: Transmitted Founder.

In order to assess whether T/F viruses are capable of infecting LCs, we first infected human skin derived activated LCs, as these have a reduced capacity to restrict HIV-1 infection, most likely due to the downregulation of langerin [4]. Notably, T/F viruses CH58, THRO and CH106 showed a significantly higher level of infection of activated LCs than the laboratory strain NL4.3Bal (Fig 1A). Next, we infected moDCs, which are susceptible to HIV-1 [5,6,14], to assess whether the T/F viruses were generally more infectious than NL4.3Bal. We observed significantly lower infection of moDCs by CH058, CH106 and THRO than the laboratory strain (Fig 1B). These data suggest that activated LCs but not moDCs are more susceptible to T/F viruses than the laboratory strain NL4.3Bal. Moreover, our data indicate that the T/F viruses are by themselves not more infectious but that this depends on the cellular background.

Human skin derived immature LCs are highly resistant to HIV-1 infection, as langerin restricts HIV-1 infection [1,2]. We therefore exposed immature LCs to T/F viruses in the *ex vivo* skin explant infection model [3] and analyzed infection of emigrated LCs. Notably, T/F viruses CH058, THRO and CH106 were significantly more efficient in infecting immature LCs ex vivo than NL4.3Bal (Fig 1C). Next we isolated and infected vaginal immature LCs with the different viruses, as these cells are highly relevant to study sexual HIV-1 transmission. Notably, T/F viruses infected vaginal LCs at higher levels than NL4.3Bal (Fig 1D). These data strongly suggest that the HIV-1 resistant LCs are more susceptible to T/F viruses.

### Immature LCs are more susceptible to T/F viruses but not to their 6-month consensus sequence virus pairs nor to laboratory-adapted strains

**Fig 2:**
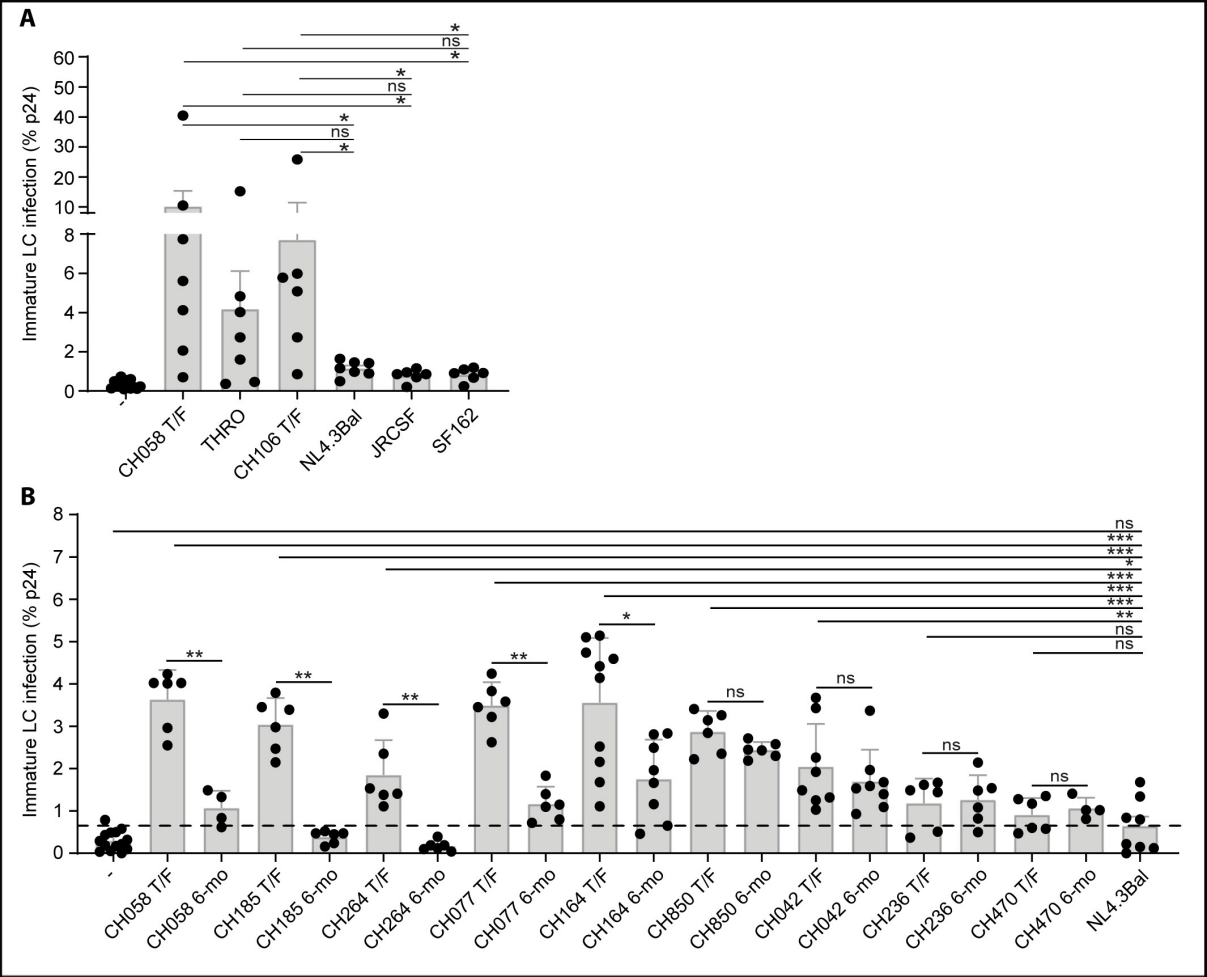
Immature LCs are more susceptible to T/F viruses but not to their 6-month consensus sequence virus pairs nor to laboratory-adapted strains. (A) Infection of immature LCs by different T/F viruses and different laboratory-adapted strains. Each dot represents the average percentage of infection per donor. Error bars are the mean ± SD of CH058, THRO, NL4.3Bal: n = 7 donors, CH106, JRCSF, SF162: n = 6 donors. (B) Infection of immature LCs by T/F viruses and their patient-matched 6-month virus pair. Each dot represents the percentage of infection per donor in duplo. Error bars are the mean ± SD of CH058, CH185, CH264, CH077, CH850, CH236, CH470: n = 3 donors, CH164: n = 5 donors, CH042: n = 4 donors. The virus titers were normalized based on RT activity. T/F: Transmitted Founder, 6-mo: 6-month consensus sequence.

We isolated immature LCs from skin and compared infection of T/F viruses with different laboratory strains. Immature LCs were infected at significantly higher levels by T/F viruses than the laboratory strains (Fig 2A). Next we treated immature LCs with HIV-1 replication inhibitors Zidovudine, Raltegravir and Indinavir to investigate whether this was due to increased HIV-1 capture or productive infection. Interestingly, infection with T/F virus CH058 was partially inhibited by the HIV-1 inhibitors (S1 Fig), suggesting that T/F viruses productively infect immature LCs as well as that these viruses are more efficiently captured by immature LCs than laboratory strains. To investigate whether the increased infection and capture of immature LCs is a general property of T/F viruses, we infected immature LCs from different donors with nine T/F viruses. Seven T/F viruses CH058, CH185, CH264, CH077, CH164, CH850 and CH042 infected immature LCs at significant higher levels than T/F viruses CH236 and CH470, and the laboratory strain NL4.3Bal (Fig 2B). The level of infection of immature LCs by CH236 and CH470 was comparable to that observed for the laboratory strains (Fig 2B). These data indicate that many but not all T/F viruses are more efficient in infecting immature LCs than laboratory-adapted strains.

Next we compared infection of immature LCs by T/F viruses and their matched 6-month consensus sequence viruses, representative of the viral quasispecies present in early chronic infection [12,13]. Notably the 6-month virus pairs from CH058, CH185, CH264, CH077 and CH164 infected immature LCs at a significant lower level than their respective T/F viruses (Fig 2B). T/F viruses CH850 and CH042 and their 6-month pairs infected immature LCs at a similar level, which was significantly higher than the laboratory strain NL4.3Bal (Fig 2B). Like their T/F counterparts, the CH236 and CH470 6-month viruses infected LCs al low levels similar to those observed for NL4.3Bal. Notably, different 6-month consensus viruses showed significantly higher levels of infection of moDCs than the CH058 T/F virus (S2 Fig), indicating that the 6-month consensus sequence viruses acquire the ability to infect moDCs. These data suggest that in subjects CH058, CH185, CH264, CH077 and CH164 viral sequence changes evolving during the first 6 months of infection, many of which are known to be driven by pressure from adaptive immune responses, resulted in a decline in the ability of the virus to infect immature LCs. In contrast, the 6-month viruses in subjects CH850 and CH042 did not undergo changes that had a similar effect on LC infection. The CH236 and CH470 6-month viruses did not acquire the ability to infect LCs at a higher level than their paired T/F viruses. These data suggest that the ability of certain T/F viruses to infect LCs might be beneficial early during infection but that this ability can be lost later in infection due to differences in immune pressure.

### T/F viruses have a lowered sensitivity to langerin restriction

**Fig 3:**
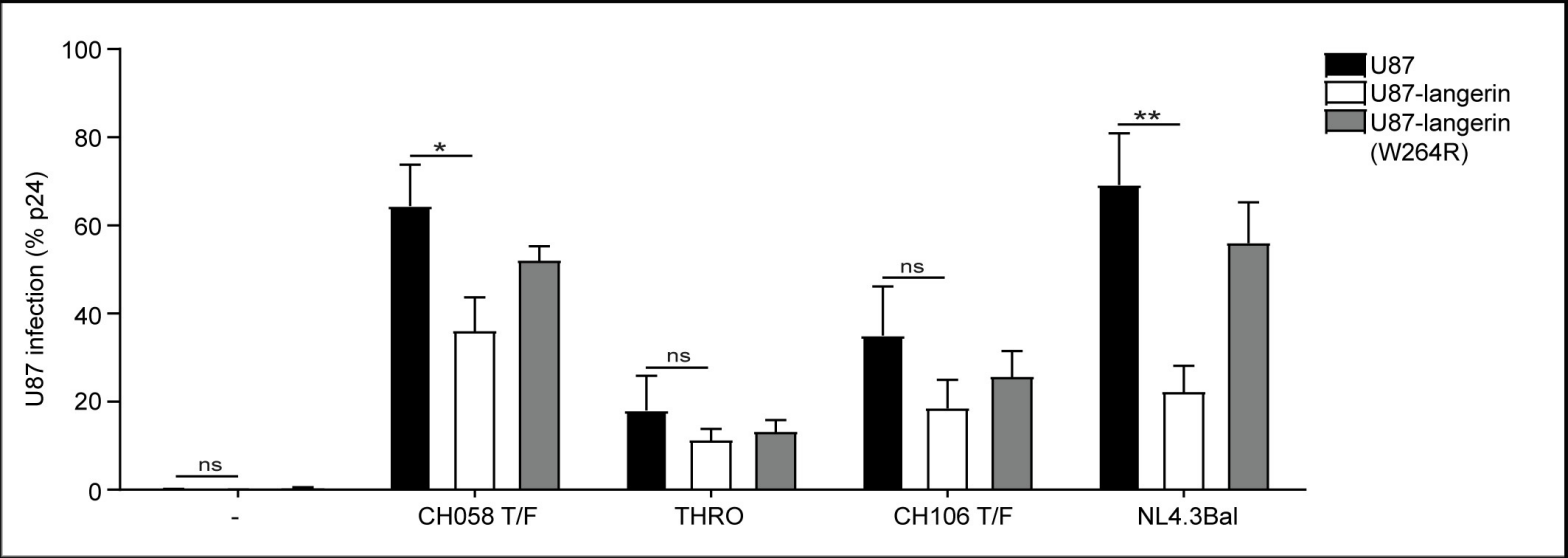
T/F viruses have a lowered sensitivity to langerin restriction. U87 cells were transduced to express langerin, or langerin with a SNP (W264R) that abrogates binding via the CRD, and infected with T/F-viruses or NL4.3Bal. Levels of infection were calculated relative to the percentage of infection in the non-transduced U87 cell line. Absolute values of infection are for U87 CH058: 64% p24, U87 THRO: 18% p24, U87 CH106: 35% p24, U87 NL4.3Bal: 70% p24. The data represents an average of 5 independent experiments, measured in duplo. Error bars are the mean ± SD of n = 5. T/F: Transmitted Founder.

To investigate whether T/F viruses are less sensitive to langerin restriction, we compared the level of infection of the highly susceptible CD4+ and CCR5+U87 cell line with that of U87 cells transduced with langerin and the langerin mutant (W264R), which is unable to bind ligands via its carbohydrate recognition domain (CRD) [2]. As shown before [2], langerin-expressing U87 cells were hardly infected by NL4.3Bal, in contrast to parental U87 cells and U87 cells expressing the langerin W264R mutant (Fig 3). Notably, langerin restriction of the T/F viruses CH058, THRO and CH106 was less efficient than that observed for NL4.3Bal. Although infection of parental U87 cells with T/F viruses THRO and CH106 was low, levels of infection of the langerin wild-type and langerin mutant expressing cells were similar. These data suggest that T/F viruses are less sensitive to langerin-mediated restriction than NL4.3Bal.

## Discussion

Sexual HIV-1 transmission is a very inefficient process. Interestingly, T/F viruses are viral variants that accomplish successful transmission, but the viral traits that facilitate this are currently incompletely understood. Here we have shown that certain T/F viruses efficiently infected LCs both in *in vitro* and *ex vivo*. As not all T/F viruses exhibited this property, these data suggest that the ability to infect LCs is not a pre-requisite for establishment of infection, but that it may confer a selective advantage during virus transmission depending on the transmission route. Comparison with donor-matched 6-month consensus sequence viruses suggested that in some individuals the T/F viruses lose this ability after establishing infection. Our data further indicate that the increased ability of certain T/F viruses to infect immature LCs is associated with a lower sensitivity to langerin-mediated restriction. Thus, the transmission success of HIV-1 might depend for certain viruses on the ability to infect LCs and therefore to circumvent LC-mediated restriction.

There are several mechanical, molecular and immunological barriers that have been shown to prevent HIV-1 transmission and infection. Local immune cells have a decisive role in the transmission process, as these cells can either protect against HIV-1 or function as initial target cells [1]. T/F viruses seem unique compared to the other viral variants in the transmission fluids in their ability to overcome local restrictive mechanisms and to become transmitted [10]. For example, T/F viruses have been shown to be relatively resistant to control by type 1 interferons, which may provide them with a replicative advantage at mucosal transmission sites and/or in draining lymph nodes [12,15]. Characterization of the phenotype of these viruses can indirectly provide insight into the decisive selective pressures *in vivo* [12,15].

Mucosal LCs restrict HIV-1 infection by targeting the viral capsid for autophagy-mediated degradation [2]. Notably, our data indicate that certain T/F viruses are relatively resistant to langerin mediated restriction in LCs. We observed efficient infection of immature LCs by certain but not all T/F viruses compared to laboratory-adapted HIV-1 strains. Interestingly, the enhanced infectivity of T/Fs compared to a laboratory-adapted virus strain observed in LCs was not paralleled in moDCs. Furthermore, several 6-month consensus sequence viruses acquired the ability to infected moDCs more efficiently than a representative T/F virus. These data further show that the superior infection of LCs by T/F viruses was not due to the presence of higher levels of virus in the T/F virus preparations or to a global difference in viral infectivity/replicative capacity, and underscores the selective ability of T/F viruses to escape restriction in LCs. Furthermore, we observed that T/F viruses were less sensitive to langerin-mediated restriction. The ability of specific T/F viruses to infect LCs might reflect their transmission route via LCs as normally LCs are restrictive to HIV-1 infection.

Notably, we observed that not all T/F viruses were efficient in infecting LCs, which strongly suggests that LC infection is not a pre-requisite for establishment of HIV-1 infection, but may confer a selective advantage during transmission, potentially by increasing the range of available transmission routes. Moreover, we observed that immature LCs are efficient in capture of and retaining T/F viruses, which suggest that T/F viruses can escape from virus degradation. Several studies have shown that LCs can either be productively infected [16,17] or capture HIV-1 [18]. Further studies into the differences between the T/F viruses might lead to a better understanding of the mechanisms by which certain T/F viruses partially escape LC-mediated restriction. For several of the T/F viruses, a matched 6-month consensus sequence virus was also generated from the same infected individual. These paired 6-month viruses are more susceptible to control by type I IFNs than their T/F counterparts [12]. Many of the sequence changes selected for in the in vivo viral quasispecies during the first 6 months of infection are driven by pressure from CD8 T cell and antibody responses [19,20]. Our data suggest that in some infected individuals, the ability to infect immature LCs is also lost during the first 6 months of infection, although we observed that two chronic pairs did not lose this ability. The 6-month viruses from subjects CH236 and CH047, whose T/F viruses did not infect LCs very efficiently, infected LCs at a similarly low level as their T/F pairs, showing that they had not acquired the ability to infect LCs during early infection. These observations suggest that although the ability to infect LC may facilitate virus transmission, it may not confer a substantial advantage on HIV-1 replication once systemic infection has been established. By contrast, HIV-1 is under intense pressure to escape from adaptive responses during acute/early infection, resulting in selection of immune escape mutations regardless of any detrimental effects on LC-infecting ability.

Comparison of the sequences of T/F viruses that infect LC efficiently with those of matched 6-month viruses from the same subjects that infect LC less well than their T/F counterparts may give insight into the mechanistic basis of enhance LC infection. Given that 6-month viruses typically contain multiple antibody-driven mutations in the viral glycoprotein, including sequence changes that alter glycosylation sites [20], it is possible that the latter may impact on langerin-binding ability, which would implicate env glycosylation-dependent effects on langerin binding as a mechanism of enhanced LC infection by some T/F viruses.

In summary, our findings strongly suggest that certain T/F viruses have the ability to infect immature LCs, a property that many viruses present during early chronic infection and laboratory virus strains lack. The ability to infect LC may confer a selective advantage on HIV-1 transmission, at least via certain transmission routes. Further detailed comparison between T/F viruses that differentially infect LCs might provide more infection into the mechanisms allowing certain T/F viruses to escape LC restriction. This study aims to aid the development of preventive measures by dissecting the events that contribute to HIV-1 transmission.

## Supporting information

S1 Fig Infection of immature LCs with CH058 T/F. Immature LCs were pre-incubated with Zidovudine (reverse transcriptase inhibitor) 20uM, Raltegravir (integrase inhibitor) 100nM, Indinavir (protease inhibitor) 1uM for 2 hours and subsequently exposed to CH058 T/F for 5 days. Cells were harvested, extensively washed, permeabilized and stained for CD1a and p24. Infection was assessed by Flow Cytometry. Each dot represents the percentage of p24 positive cells per donor in duplo. Error bars are the mean ± SD of CH058: n = 2 donors, NL4.3Bal: n = 1 donor. Statistical analysis was performed using an unpaired, non-parametric, Mann-Whitney test, *p<0.05. T/F: Transmitted Founder, inhib: inhibitor.
(TIF)

S2 Fig Infection of monocyte derived DCs with T/F virus and 6-month chronic virus. DCs were exposed to different virus strains for 5 days. Cells were harvested, extensively washed, permeabilized and stained for CD1a and p24. Infection was assessed by Flow Cytometry. Each dot represents the percentage of p24 positive cells per donor in duplo. Error bars are the mean ± SD of CH058 T/F, CH042, CH164, CH236, CH236, NL4.3Bal: n = 2 donors. Virus titers were normalized based on RT activity. Statistical analysis was performed using an unpaired, non-parametric, Mann-Whitney test, *p<0.05; ns = not significant. T/F: Transmitted Founder, 6-mo: 6-month consensus sequence.
(TIF)
