# Supplementary figures and images for: Sexually transmitted founder HIV-1 viruses are relatively resistant to Langerhans cell-mediated restriction
Source: PLoS One. 2019 Dec 19;14(12):e0226651. doi: 10.1371/journal.pone.0226651 (PMC6922402; doi:10.1371/journal.pone.0226651)

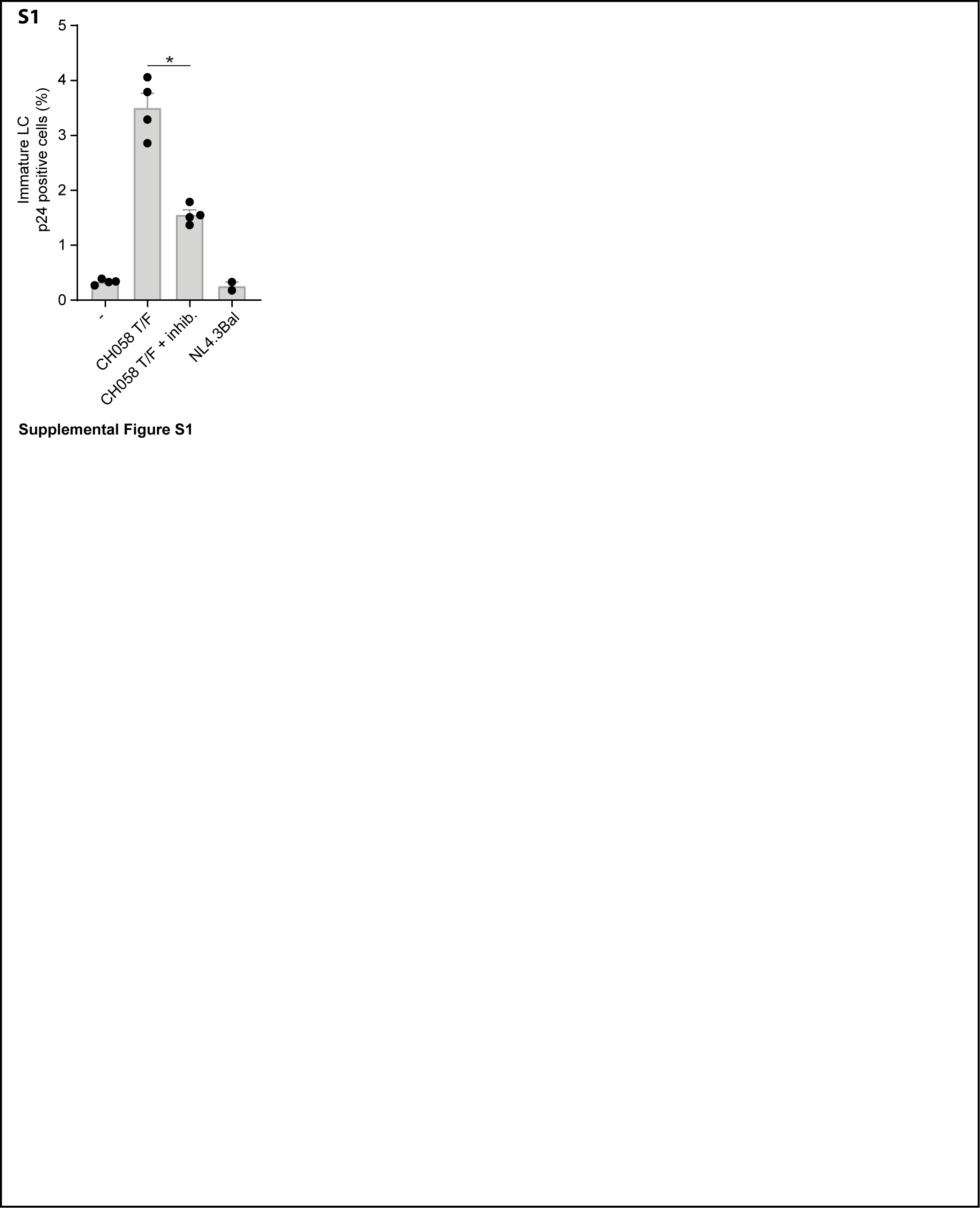

Supplement: S1 Fig — Immature LCs were pre-incubated with Zidovudine (reverse transcriptase inhibitor) 20uM, Raltegravir (integrase inhibitor) 100nM, Indinavir (protease inhibitor) 1uM for 2 hours and subsequently exposed to CH058 T/F for 5 days. Cells were harvested, extensively washed, permeabilized and stained for CD1a and p24. Infection was assessed by Flow Cytometry. Each dot represents the percentage of p24 positive cells per donor in duplo. Error bars are the mean ± SD of CH058: n = 2 donors, NL4.3Bal: n = 1 donor. Statistical analysis was performed using an unpaired, non-parametric, Mann-Whitney test, *p<0.05. T/F: Transmitted Founder, inhib: inhibitor. (TIF) [file pone.0226651.s001.tif]

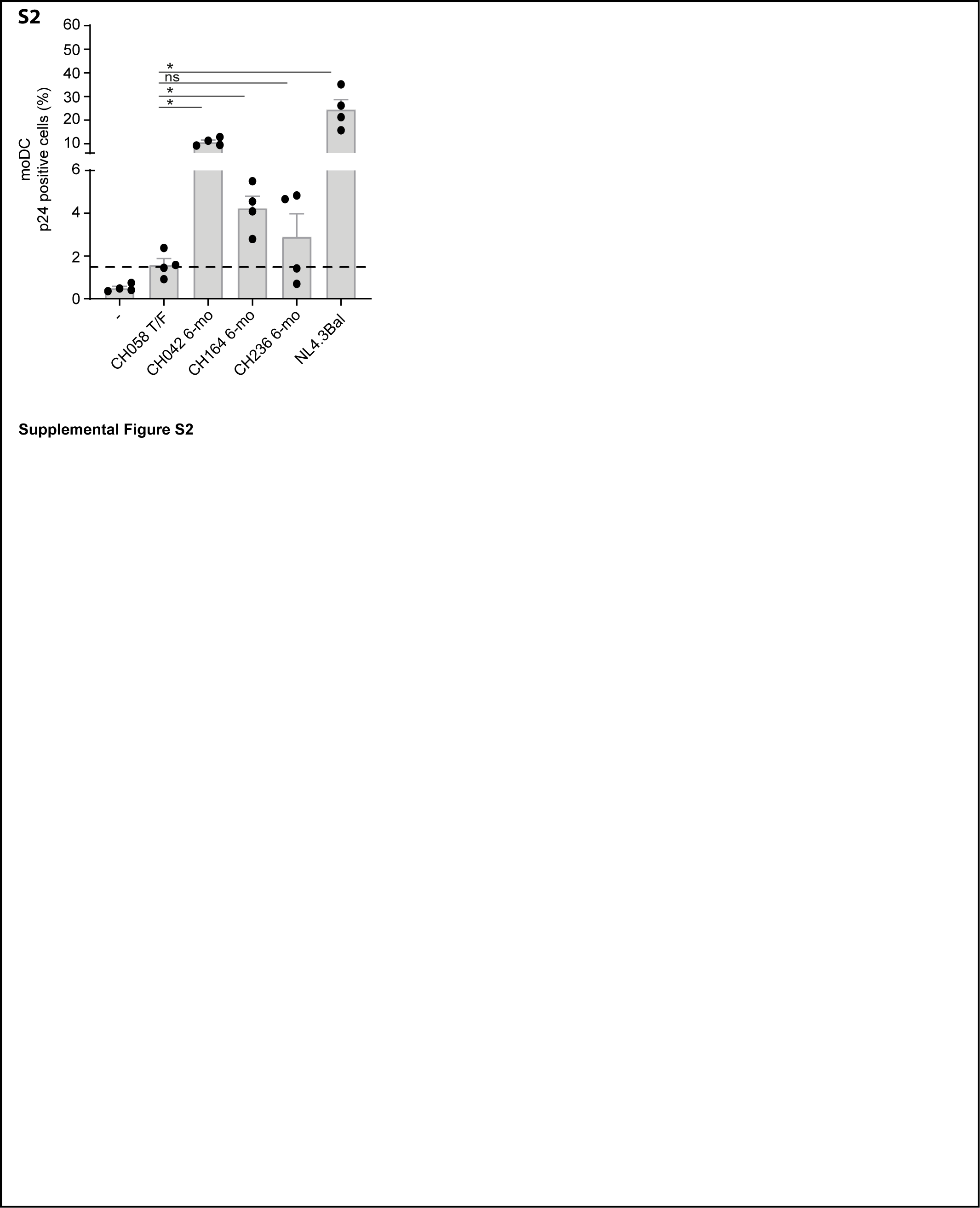

Supplement: S2 Fig — DCs were exposed to different virus strains for 5 days. Cells were harvested, extensively washed, permeabilized and stained for CD1a and p24. Infection was assessed by Flow Cytometry. Each dot represents the percentage of p24 positive cells per donor in duplo. Error bars are the mean ± SD of CH058 T/F, CH042, CH164, CH236, CH236, NL4.3Bal: n = 2 donors. Virus titers were normalized based on RT activity. Statistical analysis was performed using an unpaired, non-parametric, Mann-Whitney test, *p<0.05; ns = not significant. T/F: Transmitted Founder, 6-mo: 6-month consensus sequence. (TIF) [file pone.0226651.s002.tif]
